# Biotechnological Tools for Environmental Sustainability: Prospects and Challenges for Environments in Nigeria—A Standard Review

**DOI:** 10.1155/2012/450802

**Published:** 2012-05-03

**Authors:** Chukwuma S. Ezeonu, Richard Tagbo, Ephraim N. Anike, Obinna A. Oje, Ikechukwu N. E. Onwurah

**Affiliations:** ^1^Industrial Biochemistry and Environmental Biotechnology Unit, Chemical Sciences Department, Godfrey Okoye University, P.M.B. 01014, Enugu, Nigeria; ^2^Pure and Industrial Chemistry Unit, Chemical Sciences Department, Godfrey Okoye University, P.M.B. 01014, Enugu, Nigeria; ^3^Pollution Control and Biotechnology Unit, Department of Biochemistry, University of Nigeria, Nsukka, Enugu State, Nigeria

## Abstract

The environment is a very important component necessary for the existence of both man and other biotic organisms. The degree of sustainability of the physical environment is an index of the survival and well-being of the entire components in it. Additionally, it is not sufficient to try disposing toxic/deleterious substances with any known method. The best method of sustaining the environment is such that returns back all the components (wastes) in a recyclable way so that the waste becomes useful and helps the biotic and abiotic relationship to maintain an aesthetic and healthy equilibrium that characterizes an ideal environment. In this study, the method investigated includes biological method of environmental sustainability which seeks to investigate the various biotechnological tools (biotools) in current use and those undergoing investigations for future use.

## 1. Introduction

Biotechnological tools are those processes of bioscientific interests that use the chemistry of living organisms through cell manipulation to develop new and alternative methods aimed at cleaner and more effective ways of producing traditional products and at the same time maintain the natural and aesthetic beauty of the environment. Biotechnology is the current trend in production processes across the world, as opposed to the conventional chemical synthesis of products. The reason is due to the fact that biotechnological methods are ecofriendly while the latter method adds pollutants and waste into our environment. A lot of problems associated with conventional methods of pollutant treatment by incineration or landfills have given the impetus on the need for alternative, economical, and reliable biological methods of pollution treatments.

Chen et al. [1] enumerated vividly that environmental biotechnology refers to the utilization of microorganisms to improve environmental quality. Although the field of environmental biotechnology has been around for decades, starting with the activated sludge and anaerobic digestion in the early 20th century, the introduction of new technologies from modern microbiology and molecular biology has enabled engineers and scientists to tackle the more contemporary environment problems such as detoxification of hazardous wastes through the use of living organisms.

 As the earth's human population has increased, natural ecosystems have declined and changes in the balance of natural cycles have had a negative impact on both humans and other living systems. Thus, there is abundant scientific evidence that humanity is living unsustainably, and returning human use of natural resources to within limits will require a major collective effort [2]. Given the challenges of population increase and its attendant problems of pollution increase, biotechnology remains the most reliable means of environmental sustenance. The world is currently endangered; government and people of many counties are concerned about this endemicity of pollutants (most of which are recalcitrant) in our otherwise aesthetic environment. Africa generally and Nigeria in particular have not imbibed maximally the benefit of using biotechnology in maintenance of the beautiful environment. This paper will address the issues relating to the use of biotechnological methods vis-à-vis biotools in solving the problems of environmental degradation, with a view to encourage the adoption of these biotechnological methods in Nigeria, Africa, and other countries where waste has been a menace to the environments.

## 2. Environmental Sustainability

 Sustainability is the capacity to endure. The word sustainability is derived from the Latin *sustinere* (tenere, to hold; sus, up). In ecology the word describes how biological systems remain diverse and productive over times. For humans it is the potential for long-term maintenance of well-being, which in turn depends on the well-being of the natural world and the responsible use of natural resources (http://en.wikipedia.org/wiki/Environmental_Sustainability_Index) [3]. Environmental sustainability is the process of making sure current processes of interaction with the environment are pursued with the idea of keeping the environment as pristine as naturally possible based on ideal-seeking behaviours. An “unsustainable situation” occurs when natural capital (the sum total of nature's resources) is used up faster than it can be replenished. Sustainability requires that human activity only uses nature's resources at a rate at which they can be replenished naturally. Theoretically, the long-term result of environmental degradation is the inability to sustain human life. Such degradation on a global scale could imply extinction for humanity [4].

 A healthy environment is one that provides vital goods and services to humans as well as other organisms within its ecosystem. This can be achieved in two ways and include discovering ways of reducing negative human impact and enhancing the well-being and vitality of all living organisms (plants and animals) in the environment. Daly [5] suggested three broad criteria for ecological sustainability: renewable resources should provide a sustainable yield (the rate of harvest should not exceed the rate of regeneration); for nonrenewable resources there should be equivalent development of renewable substitutes; waste generation should not exceed the assimilative capacity of the environment.

 It is important to also clearly define what the environment is to the humans who are the focus and are adversely affected positively or negatively according to their activities within their surroundings. Thus, Bankole [6] reported that “Environment” refers to the physical surroundings of man, of which he is part and on which he depends for his activities, like physiological functioning, production, and consumption. His physical environment stretches from air, water, and land to natural resources like metals, energy carriers, soil, and plants, animals, and ecosystems. For urbanized man, a large part of his environment is man-made. But even then, the artificial environments (buildings, roads) and implements (clothes, automobiles) are the result of an input of both labour and natural resources.

## 3. Environmental Sustainability Index (ESI)

 This is a composite index tracking 21 elements of environment sustainability covering natural resource endowments, past and present pollution levels, environmental management efforts, contributions to protection of the global commons, and a society's capacity to improve its environmental performance over time [7].

 The Environmental Sustainability Index was developed and published between 1999 and 2005 by Yale University's Centre for Environmental Law and Policy in collaboration with Columbia University's Centre for International Earth Science Information Network (CIESIN), and the World Economic Forum. The ESI developed to evaluate environmental sustainability relative to the paths of other countries. Due to a shift in focus by the terms developing the ESI, a new index was developed, the Environmental Performance Index (EPI) that uses the outcome-oriented indicators, then works as a benchmark index that can be more easily used by policy makers, environmental scientists, advocates and the general public [8].

## 4. The Nigerian Physical Environment

### 4.1. The Niger Delta Environment

Nigeria has one of the worst environmental records in the world. In late 1995, Nigeria's execution of eight environmental activists, notable Nobel Peace Prize nominee Ken Saro-Wiwa, made international headlines and brought world-wide recognition of the serious environmental degradation of Nigeria [9]. Today, the oil-rich Niger Delta region of Nigeria is always the first point of reference when analysing the Nigerian environment. This stems from the fact that despite the sacrifice of Ken Saro-Wiwa and others, much has not changed in terms of making the environment pollution free. In fact, it has even gotten worse with time and recent developments. During the 1990s, the Niger Delta locals learned that extortion pays. Villagers found that by sabotaging oil installations to collect oil spill compensation from shell (an oil firm) they could earn more than by marginal subsistence farming on degraded lands. Thus, sabotaging and spills became a new dimension of increasing crude oil pollutants. Attacks on oil facilities and pipelines became even more relentless, and the Niger River delta was an increasingly bloody place. Environmental degradation from crude oil productions continued, and by 1999 the United Nations declared the delta the most threatened in the world [9]. In early 2006, conditions worsened in the delta. The number of kidnapping of oil workers increased as did attacks on oil facilities. The Niger Delta is made up of six states of the south-south region, namely, Bayelsa, Akwa Ibom, Cross River, Delta, Rivers, and Edo.

 This lingering problem caused the former President of Nigeria, Alhaji Umaru Musa Yar'adua, to create a Ministry of the Niger Delta so as to oversee the well-being of the environment and the people of this region. Currently more is being done in the Niger Delta to bring about reduction in vandalization of oil pipelines as well as an amnesty for the irate youths in this part of Nigeria in order to find a lasting solution to both the social and environmental problem of the Niger Delta area of Nigeria.

 Crude oil spill affects germination and growth of some plants [10], it also affects the overall production of crop (e.g., *Zea mays*) due to its negative impact on the chlorophyll content which is a marker of the yield of plants [11]. Severe crude oil spill in Cross River state, Nigeria, has forced some farmers to migrate out of their traditional home, especially those that depend solely on agriculture. The negative impact of oil spillages remains the major cause of depletion of the Niger Delta of Nigeria vegetation cover and the mangrove ecosystem [12].

### 4.2. The Nigerian Environment: Case Study of Solid Waste Generation in Cities

The Nigerian cities such as Aba, Enugu, Onitsha, Kano, Ibadan, and Lagos are characterised by huge mounds of solid waste dumps generated from households, industries, markets, schools, and street trading. This can be attributed to migration, population increase, urbanization, constructions, and industrialization coupled with inefficient, improper and some times nondisposal of wastes. Solid waste dumps are indiscriminately formed on streets, homes, road side, markets, and other places where human activities take place in the cities.

 Solid wastes can be broadly grouped into two as it relates to the concept of this write up. These two categories are the following.

**(a) The Biodegradables (Biowastes)**

These include those solid wastes generated, which could be decomposed by microorganisms and does not constitute major sources of pollution for a long period of time. They are paper products (such as printing papers, waste books, newspapers, carton, toilet paper, card boards), and wastes of plant origin (fruits, stems, roots, vegetables, leaves, food remains and garden solid wastes, etc.), wastes of animal origin (faecal matter, carcass, droppings, and poultry waste products). These groups of solid waste even though they are easily degraded by microorganism in minimal time, give off offensive odour and constitute nuisance to the aesthetic environment more than the nonbiodegradable solid wastes. They can also constitute a good habitat for the thriving of pathogenic microorganisms which could easily pollute fresh food product and sources of fresh water in the urban cities in Nigeria.

**(b) Nonbiodegradable (Rubbish/Garbage)**

These groups of solid wastes are not degradable or hardly degraded by microorganisms. Hence, other means of treatment such as incineration, land refill, and recycling are currently employed in Nigeria as ways of disposing them. Examples of this group of solid wastes are solid wastes of metallurgical and smelting industries (abandoned vehicles, motor cycles, vehicle part and scrap metals, iron, zinc, aluminium sheets and other metals, machine parts); solids wastes of construction industries (sand, gravel, bitumen wastes, concrete and waste building materials); solid waste of plastic industries (plastic buckets, cable insulators, tyres, chairs, tables, cellophane bags, plastic bottles, cutleries, sachet water containments, etc.) and glass products. These might not give out offensive odour, but they are even worse nuisance to the environment since their disposal has become a “Herculean” and near-impossible task in Nigeria.

Solid waste management activities include prevention (pollution prevention from sources), source reduction (pollution minimization in waste generating activities at point of good production), and treatment (safe disposal of nonrecyclable residues, recycling, transport of waste to land refills).

The major problem is that Nigeria is yet to develop efficient ways of waste disposal which are eco-friendly and which could be recycled back into the environment without constituting nuisance to the environment or affecting the health of the biotic components of the ecosystem.

### 4.3. Environmental Degradation due to Mining Activities in Nigeria

 The natural topography of many cities and country side in Nigeria had been destroyed as a result of commercial activities involved in the exploration and exploitation of numerous minerals that abound in the country. Places like Jos, Bauchi, Nasarawa, and Enugu states have been worst affected by environmental degradation which had defaced the beautiful landscape of the natural environment.

 No consistent mining regulatory law is enforced in the country. The exploration of tin in the Plateau (Jos) started as early as 1808 by the British colonialists, and in the 1970s Nigeria produced an average of 10,000 tons of tin ore annually. Output fell to 3,000 tons in the 1980s and dropped again to 500 tons in the 1990s. Nigeria now earns less than 0.5% of its foreign exchange from tin [13]. For over 70 years Jos tin mining industry was mostly controlled by overseas companies. But when the company was nationalised in 1972, no one took responsibility for clearing up the mess left behind. In places on the plateau such as Bukuru, Rayfield, Barkin Ladi, Mangu, Anglo Jos, Zawan, Du, Shen, Gyel and Shere Hills, ugly gashes left over from past mining activities can be seen everywhere. Alarmingly, effluents from nearby industries have seeped deep into mines-turned-water holes. Farmers use water from dams which resulted from tin mining activities for irrigation. The top soil also washes into streams in neighbouring village water that is used for drinking and other domestic purposes.

 In addition, locals use soil left over from the abandoned mining sites—containing naturally found radioactive heavy metals to build houses. Environmentalists fear that people living in these houses risk being exposed to unhealthy levels of radiation [13].

 Tin mining has also displaced many people from fertile agricultural land. The mining sites are located in the best areas in terms of the terrain and the flatness of the land. The people are now compelled to farm on rocky land. Government on its part instead of reclaiming the lands and resettling the people only asked people whose lands are destroyed to move out of these danger zones without compensation or arrangements to resettle them properly.

 Thus, the tin areas have environment whose topography is made up of dams (which claim lives of both human and animals annually) as well as ‘‘a lunar landscape of steep-sided mounds with multicoloured ponds or lakes” [13].

 Nigerian environmentalists have agreed that mining activities such as tin-Jos, Coal-Enugu, and others have done great damages to the environment which will need a concerted effort especially adoption of better mining practices in order to remediate.

### 4.4. Erosion, Desertification, and Deforestation: Loss of Biodiversity in the Nigerian Environment

Erosion problem is also a major environmental threat in Nigeria as sheet and gully erosion have wrecked untold havoc in several states such as Abia, Adamawa, Anambra, Delta, Ebonyi, Edo, Enugu, Gombe, Jigawa, Kogi, Ondo, Ogun, and Lagos. In Lagos state and other coastal areas, coastal erosion has destroyed properties and valuable lands were washed away. Most of the flooding and erosion seen in cities are as a result of poor drainage system.

 Places such as the eastern states of Nigeria (Anambra, Imo, Abia, Enugu, and Ebonyi) have regions prone to erosion. This has resulted in the entire loss of farm land and buildings. The situation is so pathetic that a whole clan in a southern part of Anambra state was forced to take refuge in a primary school. Places like Agulu and most part of Aguata and Orumba Local government areas are highly endangered with erosion invasion. The Northern part of the country has ecosystem characterised by the Savannah clime. Starting from the North Central region encompassing Benue to Katsina states in the farthest part of the North made up of the southern savannah, Northern savannah, Sudan, and Sahel savannah characterized by low foliage and little trees. The environment is marked by constant grazing and building of huts which affect the type of plant survival as well as desert encroachment from the Niger and the Chad republics at the furthest part of the country.

 Deforestation is a serious problem in Nigeria, which currently has one of the highest rates of forest loss (3.3 Percent) in the world. Since 1990, the country has lost some 1 million hectares or 35.7 percent of its forest covers [9]. Worse Nigeria's most biodiverse ecosystems—its old-growth forests is that are disappearing at an even faster rate. Between 1990 and 2005, the country lost a staggering 79% of these forests and since 2000 Nigeria has been losing an average of 11 percent of its primary forests per year—double the rate of the 1990s. These figures mark Nigeria as having the highest deforestation rate of natural forest on the planet. As its forests fall, Nigeria has seen wildlife populations plummet downward from poaching and habitat loss, increasing desertification. It appears that Nigeria's swift economic development has exacted a high toll on its people and environment [9].

 The problems of environmental degradation have continued to plague Nigeria, and they have defied proffered solution mainly due to improper applications and also the lack of proper waste control and environmental maintenance. The major causes of environmental degradation problems were identified by the Vision 2010 Committee set up by the Federal Government. Aina and Salau [14] enumerated some of these problems as follows:

- poverty as a cause consequence of environmental exploitation, with the poor scavenging marginal lands to eke out a living;
- bush burning for farming and ever-increasing depletion of young forests for fuel wood.
- uncontrolled logging accentuated by lack of re-stocking in many parts of the country. This practice is linked with the loss of precious biological diversity (nature's gene bank of raw materials for future development);
- gas flaring, Crude oil spill and the resultant problem of ecosystem destabilization, heat stress, acid rain and acid precipitation-induced destruction of fresh water fishes and forests in the coastal areas of the country. Nigeria alone accounted for about 28% of the world's total gas flared;
- a general inability of the agencies responsible for the environment to enforce laws and regulations, particularly with respect to urban planning and development, prospecting for minerals and adherence to industrial standards, sitting of public and residential quarters in flood-prone areas, unsettled dump site improperly reclaimed and converted to plots.

## 5. The Need for Pollution Prevention

 Most of the pollutants in the environment are directly or indirectly the product of industrial activities/production. Awareness of the deleterious effect of pollutants in the environment is on the increase. Government, environmentalists, and communities for a long time have been frowning at the degradation of the environment due to man-made pollutants especially those that are by-product of industries. Industries on the other hand are under pressure by their communities to minimize the pollutants they generate. This has placed the manufacturing industries at high cost of revenue for pollution treatment as well as Billion of Naira for research into eco-friendly ways of manufacturing processes which minimizes pollution generation. Most of the pollution released from industrial processes includes discharge into the environment, namely: air, land and water. The best points of pollution prevention involves, source reduction (by using raw materials more efficiently); pollution control (substituting less harmful substances for hazardous materials); pollution management (eliminating toxic substances from the production process).

 By implementing pollution prevention practices, companies often reduce their operational waste disposal, and compliance costs (http://www.p2.org/about/nppr_p2.cmf/).

## 6. Biotechnology: The Hope for Environmental Sustainability

 As earlier stated, man's activities in his environment involve a lot of chemical synthesis in the process of converting the natural products in his environment into other forms convenient for his consumption. In the quest for converting wood into timber, use of fruits in juice production, use of herb for drug synthesis, conversion of petrochemical substances into polythene products, the environment correspondingly becomes littered with substances not needed in the cause of production. In the process of creating products, man also creates problems either consciously or unconsciously vis-à-vis pollution. As a result, the most acceptable solution to the generated wastes in the environment is such that will conveniently integrate them back into the environment. That method involves the use of microorganisms—usually yeasts, bacteria, or fungi as whole cell usage production system or in the form of industrial enzymes. In many cases these microorganisms or their products are integrated into the substrates which give us the products, desired in the industries, examples of these are bioleaching (biomining), biodetergent, biotreatment of pulp, biotreatment of wastes (bioremediation), biofiltrations, aquaculture treatments, biotreatment of textiles, biocatalysts, biomass fuel production, biomonitoring, and so forth. These are biotools (biotechnological tools), which could solve the problem of pollution and help sustain the environment. This is so because when the products or their constituents are discarded, they go back into the ecosystem. As such, they become reconverted into organic components of the environments. Moreover, their production is strictly biological instead of chemical (synonymous to pollution introduction).

 These biotechnology tools have long been used in many developed countries in the world such as the United States, Finland, Sweden, Germany, Japan, and others. Africa is still lagging from being integrated into these environmental sustainability best practices. Nigeria is the focus on how to begin to make use of these biotools for the improvement of the badly degraded environment.

## 7. Biotechnology

 Biotechnology is defined as a set of scientific techniques that utilize living organisms or parts of organisms to make, modify, or improve products which could be plants or animals. It is also the development of specific organisms for specific application or purposes and may include the use of novel technologies such as recombinant DNA, cell fusion, and other new bioprocesses [15].

 Biotechnology is not new; it has been employed for centuries in the production of fermented foods such as gari, bread, yoghurt, and cheese and beverages such as wine and beer [16]. Thus, it is a natural phenomenon in use even in Africa (Nigeria) though its principle was not well understood. CTA [16] report illustrated the denomination of “green,” “red” and “white” biotechnology according to its uses and applications. Moreover, Disilva [17] has a different classification as shown in Table 1.

Despite the that classification for convenience, using the CTA [16] classification encompasses all others. As a result “Green biotechnology” encompasses a wide range of techniques that consists of culturing plant tissues and/or organs, followed by the multiplication of the relevant plants with desirable characteristics. Genetically identical plantlets are thus available for distribution to farmers, horticulturalists, forestry growers, and nurseries all the year round. It also includes the transformation of plants, crop species, and varieties through genetic engineering techniques, leading to what are known as “genetically modified” (GM) crops. In addition, green or agricultural biotechnology also applies to techniques used in livestock husbandry (nutrition and reproduction). Green biotechnology should therefore not only be equated with advances in genetic engineering.

“Red biotechnology” encompasses the genetic engineering technique that has been used since the mid-1970s to produce drugs and vaccines in microorganisms, animal cells, and more recently in plants. For example, insulin, human and bovine growth hormones, interferon, cell growth factors, antihepatitis B vaccine, and others are being produced in this way. A wide range of diagnostic techniques and veterinary vaccines are produced using red or medical biotechnology [16].

 “White biotechnology” refers to a wide range of processes resulting in fermented products and chemicals (e.g., enzymes, biofuels such as ethanol and bioplastics) as well as to the technologies used in recycling waste water, industrial effluents, and solid wastes. These “bioremediation” processes contribute to the abatement of pollution. The extraction of metals from ores with the help of microorganisms (biomining) is also part of white or environmental biotechnology [16].

## 8. Environmental Biotechnology

 Environmental biotechnology is “the integration of natural sciences and engineering in order to achieve the application of organisms, cells, parts thereof and molecular analogues for the protection and restoration of the quality of our environment” [18].

 The nomenclature of “white biotechnology” is alluded to both industrial and environmental biotechnology. Biotechnological tools for environmental sustainability are qualified to be greatly associated as major component of the “White biotechnology.”

 Biotechnological processes to protect the environment have been used for almost a century now, even longer than the term “biotechnology” exists [18]. Municipal sewage treatment plants and filters to purify town gas were developed around the turn of the century. They proved very effective although, at the time, little was known about the biological principles underlying their function. Since that time, our knowledge base has increased enormously [18].

 Biotechnological techniques to treat waste before or after it has been brought into the environment are components of environmental biotechnological tools. Biotechnology can also be applied industrially for use in developing products and processes that generate less waste and use less nonrenewable resources and consume less energy. In this respect biotechnology is well positioned to contribute to the development of a more sustainable society through a sustainable environment. Recombinant DNA technology has improved the possibilities for the prevention of pollution and holds a promise for a further development of bioremediation [18]. What this means for environmental biotechnology is that it is futuristic and limitless in application and usage.

## 9. Biotechnological Tools for Environmental Sustainability

 Biotools for the sustenance of the environment are those biotechnological processes that make use of bioproducts as well as microorganisms for pollution reduction, production of environmental friendly products as well as general maintenance of the pristine (natural) environment for the benefit of man and other ecosystem components. It is an aspect of environmental biotechnology concerned with prevention of processes capable of causing an unsustainable environment for man and ecocomponents. Some of the biotools in use will be briefly and concisely enumerated here, and it is by no means exhaustive due to current and future addition to the body of knowledge in the environmental biotechnology field. The discussion will centre on the current or future projection of the usage of these tools elsewhere and the need for Nigeria and other countries which are hitherto not adapting to their usage due to environmental and technological limitations to break such barriers and begin in earnest to adopt their usage. Some of the biotools are as enumerated in the following.

### 9.1. Biodetergents and Biosolvent Research/Production

FAIR [19] stated that solvents and detergents are important in a number of industries, and most are derived from petroleum. There is increasing concern that prolonged contact with solvents causes health problems for workers in the factories where solvents and detergents are produced and for those using such substances in domestic and commercial laundry in their everyday work. The aim for research into biodetergents is to create biological substitutes for solvents and detergents derived from petroleum. Apart from detergents, research is also ongoing into developing substitutes of biological origin for solvents used in the production of paints, offset-printing ink, and so forth. These new solvents will be made by mixing together several common bioliquids: such as bioethanol, terpenes, vegetable oils, fatty acids, methyl esters, and derivatives of related compounds [19].

 The key to success is mixing the right compounds together in the right proportions. To do this, researchers are developing mathematical models, which will allow the “recipes” for the new biosolvents to be optimised. The mixtures being developed are designed to meet the criteria established by the companies participating in the project. Those small- and medium-sized enterprises from France, the United Kingdom, The Netherlands, Denmark, and Belgium are paint producers, manufacturers of ink for use in offset printing, and producers of detergent. Their activities are examples of only three industries where solvents are used: the application of biosolvents to other industrial processes will also be promoted as part of this project [19].

#### 9.1.1. Benefits for Farmers, Workers, and the Environment

 FAIR [19] SMEs research release reported that most solvents and detergents in current use are derived from petroleum, as a result there are two major drawbacks to these solvents and detergents compared to the biological counterparts. First, one day the crude oil from which they are derived will no longer be available since they are nonrenewable natural resources and again they present a major waste disposal problem as they do not break down readily when disposed of hence pollute the environment.

 Presently [19], the success of the new biosolvents and detergents are limited since they are more expensive than the traditional products. At the moment, conventional solvents are cheap and the replacement bioproducts could not compete on price. But as governments start to use taxation to stimulate the use of renewable resources, economic viability of biosolvents should improve. Increased use of biosolvents and biodetergents will stimulate demand for the raw materials used to manufacture the new products and so help farmers by creating a new outlet for their produce. The new solvents and detergents being developed will have a much less detrimental effect than existing products on the health of workers making and using them. Agricultural product, job creation, and environmental sustainability are the benefits accruable due to this biotool innovation. Development of the product will help lessen dependence on non-renewable resources (prevents nonrenewable environmental exploitation), safeguard human health, and protect the environment from chemical pollutants seeping into aquatic body and atmosphere.

 Nigeria can take a cue from this giant stride and begin its own processing of the natural and agricultural product as well as make use of the fertile land to develop bio products friendly to the environment. It is a viable project friendly to the environment. It is a viable project which can be adopted since it requires less energy consumption and minimal technological effort. Hence, Nigerian government, environmentalists, industrialists, and research institution can adopt this novel production of biodetergent and biosolvents for its environmental sustainability.

### 9.2. Bioremediation

 Bioremediation is the use of biological systems for the reduction of pollution from air or from aquatic or terrestrial systems [18], it also involves extracting a microbe from the environment and exposing it to a target contaminant so as to lessen the toxic component [20]. Thus, the goal of bioremediation is the employment of biosystems such as microbes, higher organisms like plants (phytoremediation) and animals to reduce the potential toxicity of chemical contaminants in the environment by degrading, transforming, and immobilizing these undesirable compounds.

 Biodegradation is the use of living organisms to enzymatically and otherwise attack numerous organic chemicals and break them down to lesser toxic chemical species. Biotechnologists and bioengineers classify pollutants with respect to the ease of degradation and types of processes that are responsible for this degradation, sometimes referred to as treatability [20].

 Biodegradation with microorganisms is the most frequently occurring bioremediation option. Microorganisms can break down most compounds for their growth and/or energy needs. These biodegradation processes may or may not need air. In some cases, metabolic pathways which organisms normally use for growth and energy supply may also be used to break down pollutant molecules. In these cases, known as cometabolisms, the microorganism does not benefit directly. Researchers have taken advantage of this phenomenon and used it for bioremediation purposes [18].

 A complete biodegradation results in detoxification by mineralising pollutants to carbon dioxide (CO_2_), water (H_2_O), and harmless inorganic salts [18]. Incomplete biodegradation (i.e., mineralization) will produce compounds that are usually simpler (e.g., cleared rings, removal of halogens), but with physical and chemical characteristics different from the parent compound. In addition, side reactions can produce compounds with varying levels of toxicity and mobility in the environment [20].

 Biodegradation may occur spontaneously, in which case the expressions “intrinsic bioremediation” or “natural attenuation” are often used [18]. In many cases the natural circumstances may not be favourable enough for natural attenuation to take place due to inadequate nutrients, oxygen, or suitable bacteria. Such situations may be improved by supplying one or more of the missing/inadequate environmental factors. Extra nutrients [18] were disseminated to speed up the break down of the oil spilled on 1000 miles of Alaskan shoreline by the super tanker Exxon Valdez in 1989.

 According to Vallero [20], there are millions of indigenous species of microbes living at any given time within many soil environments. The bioengineer simply needs to create an environment where those microbes are able to use a particular compound as their energy source. Biodegradation processes had been observed empirically for centuries, but putting them to use as a distinct field of bioremediation began with the work of Raymond et al. [21]. This seminal study found that the addition of nutrients to soil increases the abundance of bacteria that was associated with a proportional degradation of hydrocarbons, in this case petroleum by-products [21].

#### 9.2.1. Life Chemical Dynamics (Biochemodynamics) of Bioremediation

 Bioremediation success [20] depends on the following:

1. the growth and survival of microbial populations; and
2. the ability of these organisms to come into contact with the substances that need to be degraded into less toxic compounds;
3. sufficient numbers of microorganisms to make bioremediation successful;
4. the microbial environment must be habitable for the microbes to thrive.

Sometimes, concentrations of compounds can be so high that the environment is toxic to microbial populations. Therefore, the bioengineer must either use a method other than bioremediation or modify the environment (e.g., dilution, change of pH, pumped Oxygen, adding organic matter, etc.) to make it habitable. An important modification is the removal of non-aqueous-phase liquids (NAPLs) since the microbes' biofilm and other mechanisms usually work best when the microbe is attached to a particle; thus, most of the NAPLs need to be removed, by vapour extraction [20]. Thus, low permeability soils, like clays, are difficult to treat, since liquids (water, solutes, and nutrients) are difficult to pump through these systems. Usually bioremediation works best in soils that are relatively sandy, allowing mobility and greater likelihood of contact between the microbes and the contaminant [20]. Therefore, an understanding of the environmental conditions sets the stage for problem formulation (i.e., identification of the factors at work and the resulting threats to health and environmental quality) and risk management (i.e., what the various options available to address these factors are and how difficult it will be to overcome obstacles or to enhance those factors; that make remediation successful). In other words, bioremediation is a process of optimization by selecting options among a number of biological, chemical and physical factors these include correctly matching the degrading microbes to conditions, understanding and controlling the movement of the contaminant (microbial food) so as to come into contact with microbes, and characterizing the abiotic conditions controlling both of these factors [20]. Optimization can vary among options, such as artificially adding microbial populations known to break down the compounds of concern. Only a few species can break down certain organic compounds [20]. Two major limiting factors of any biodegradation process are toxicity to the microbial population and inherent biodegradability of the compound. Numerous bioremediation projects include in situ (field treatment) and ex situ (sample/laboratory treatment) waste treatment using biosystems [20].

Table 2 shows the application of bioremediation in various environmental processes.

## 10. A Practical Application of Microorganism in Crude Oil Bioremediation

 According to Onwurah [22] many microorganisms can adapt their catabolic machinery to utilize certain environmental pollutants as growth substrates, thereby bioremediating the environment. Some microorganisms in carrying out their normal metabolic function may fortuitously degrade certain pollutants as well. This process termed cometabolism obviously requires adequate growth substrates. Diazotrophs, such as *Azotobacter vinelandii*, beyond their ability to fix atmospheric nitrogen also have the capacity, in some case, to cometabolise petroleum hydrocarbons [10].

Onwurah [22] carried out a bioremediation study that involved two bacteria, a hydrocarbonoclastic and diazotrophic bacteria. The hydrocarbonoclastic was tentatively identified as *Pseudomonas *sp. and designated as NS_50_C_10_ by the Department of Microbiology, University of Nigeria, Nsukka. The diazotrophic bacteria was *Azotobacter vinelandii*, which was isolated from previously crude oil-contaminated soil [10]. This study describes the mineral media and procedure for isolation and multiplication of the bacteria to the required cell density. Crude oil spill was simulated by thoroughly mixing 50, 100, and 150 mg fractions of crude oil with 100 g batches of a composite soil sample in beakers. The soil samples were taken from a depth of 0–50 cm from the Zoological garden, University of Nigeria, Nsukka. The mixing was conducted using a horizontal arm shaker adjusted to a speed of 120 rpm for 30 minutes. The contaminated soil samples, in beakers, were inoculated with optimal combinations (cell density) of NS_50_C_10_ and *A. vinelandii*. Water was added to the crude oil-contaminated soil samples (both inoculated and those not inoculated to a saturation point but not in excess), and then the samples were left to stand undisturbed for seven days. NS_50_C_10_ was applied first, followed by *A. vinelandii*, 12 hours later. At the seventh day of soil treatment, 20 sorghum grains (previously soaked overnight in distilled water) were planted in each soil sample followed by irrigation to aid germination. Seven days after the planting of the sorghum grains, the soil from each beaker was carefully removed. The number of germinated seed per batch of soil sample was noted, the length of radicules was measured, and the mean length was taken from each batch.

The results of this experiment showed that *Pseudomonas *sp. grew well on agar plates containing a thin film of crude oil as the only carbon source, while *A. vinelandii* did not. However, cell-free extract of *Azotobacter vinelandii* fixed atmospheric nitrogen as ammonium ion (NH_4_^+^) under appropriate condition. The specific growth rate values in contaminated soil samples inoculated with both normal NS_50_C_10_ and *A. vinelandii* (consortium) were highest in all cases. By adding an aerobic, free living diazotroph *A. vinelandii* with the *Pseudomonas sp.* (NS_50_C_10_), an improvement on bioremediation of soil over that of the pure NS_50_C_10_ alone was achieved to the order of 51.96 to 82.55%. This innovative application that uses the synergetic action of several microorganisms to clean up oil-polluted soil has potential application for the bioremediation of oil-contaminated soil in the Niger delta region.

**Figure 1:**
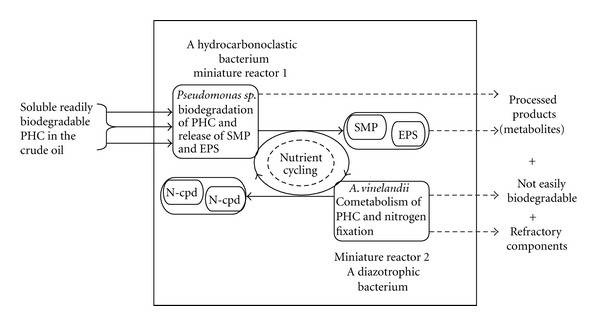
Simplified bioremediation conceptual model of *Pseudomonas *sp. and *A. vinelandii* operating as a unit of two miniature sequencing bioreactors, in situ (SMP: soluble microbial products; N-cpd: fixed nitrogen compounds; EPS: exopolysaccharide; PHC = petroleum hydrocarbons) [22].

The method described above is the biotechnological application known as *bioaugmentation *which is the addition of selected organisms to contaminated soils (sites) in order to supplement the indigenous microbial population and speed up degradation.  Figure 1 presents a model of the process involved in this bioremediation technique.

This bioremediation method by the authors has been applied in bioremediation especially in Niger delta areas of Nigeria. The authors also serve in the capacity of industrial consultants in the specialized field of crude oil pollution clean-up procedures using this specific biotool (bioremediation).

### 10.1. Biofiltration

 This is a pollution control technique employing the use of living material to capture and biologically degraded process pollutants. Common uses of biofiltration processes are for processing waste water, capturing harmful chemicals or silt from surface runoff, and microbiotic oxidation of contaminants in air (http://www.biofilter.com/).

 In multimedia-multiphase bioremediation, waste streams containing volatile organic compounds (VOCs) may be treated with combinations of phases, that is, solid media, gas, and liquid flow in complete biological systems. These systems are classified as three basic types: biofilters, biotrickling filters, and bioscrubbers (http://www.biofilter.com/). Biofilms of microorganisms (bacteria and fungi) are grown on porous media in biofilters and biotrickling systems. The application of this biotechnological tool includes the following.

#### 10.1.1. Control of Air Pollution

**Figure 2:**
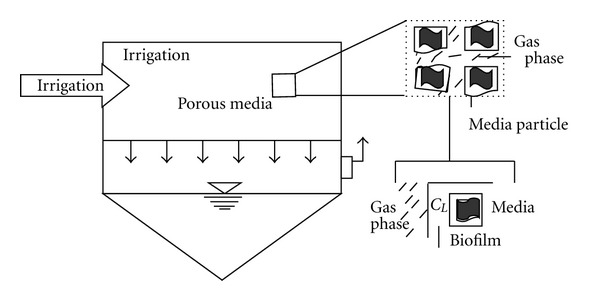
Schematic of packed bed biological control system to treat volatile compounds. Air containing gas phase pollutants (CG) traverse porous media. The soluble fraction of the volatilized compounds in the air steam partition into the biofilm (CL) according to Henry's Law. CL = {CG/H} where H is Henry's Law constant. Adapted and modified from Vallero [23].

When applied to air filtration and purification, biofilters use microorganisms to remove air pollution (http://www.biofilter.com/). The air flows through a packed bed, and the pollutant transfers into a thin biofilm on the surface of the packing material. Microorganisms, including bacteria and fungi, are immobilized in the biofilm and degrade the pollutant. Trickling filters and bioscrubbers rely on a biofilm and the bacterial action in their recirculating waters (http://www.biofilter.com/). The air or other gas containing the VOCs is passed through the biologically active media, where the microbes break down the compounds to simpler compounds, eventually to carbon dioxide (if aerobic), methane (if anaerobic), and water. The major difference between biofiltration and trickling systems is how the liquid interfaces with the microbes. The liquid phase is stationary in a biofilter (Figure 2), but liquids move through the porous media of a biotrickling system (i.e., the liquid “trickles”).

**Figure 3:**
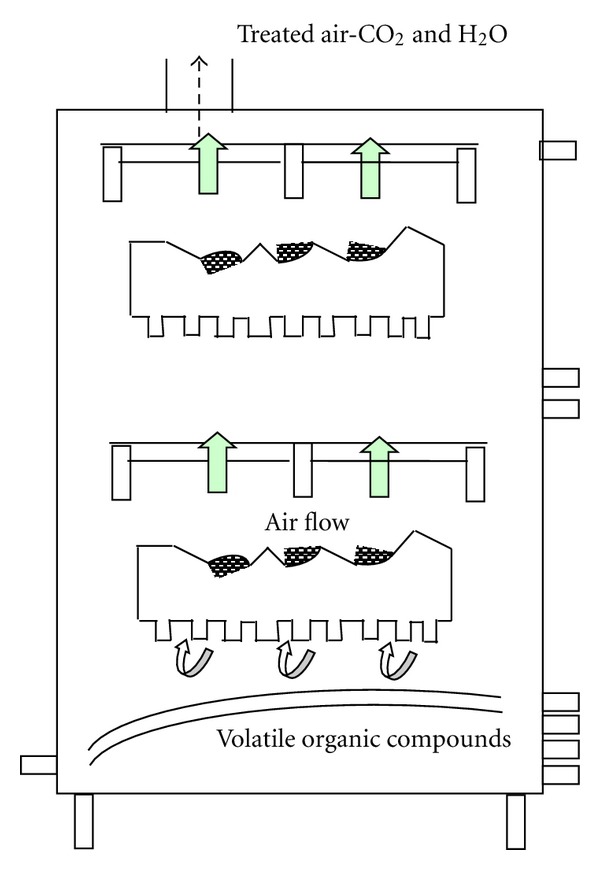
Biofiltration without a liquid phase used to treat vapour phase pollutants. Air carrying the volatilized contaminants upward through porous media (e.g., compost) containing microbes acclimated to break down the system can be heated to increase the partitioning to the gas phase. Microbes in the biofilm surrounding each individual compost particle metabolize the contaminants into simpler compounds, eventually converting them into carbon dioxide and water vapour. Modified from Vallero, [20].

A particular novel biotechnological method in biofilteration (Figure 3) uses compost as the porous media. Compost contains numerous species of beneficial microbes that are already acclimated to organic wastes. Industrial compost biofilters have achieved removal rates at the 99% level [20]. Biofilters are also the most common method for removing VOCs and odorous compounds from air streams.

In addition to a wide assortment of volatile chain aromatic organic compounds, biological systems have successfully removed vapour-phase inorganics, such as ammonia, hydrogen sulfide, and other sulfides including carbon disulfide, as well as mercaptans. The operational key is the biofilm. The gas must interface with the film. Compost has been a particularly useful medium in providing this partitioning [20]. Industries employing the biofiltration technology include food and animal products, off-gas from waste water treatment facilities, pharmaceuticals, wood products manufacturing, paints, and coatings application and manufacturing and resin manufacturing and application. Compounds treated are typically mixed VOCs and various sulfur compounds, including hydrogen sulfide (http://www.biofilter.com/). Maintaining proper moisture condition is an important factor in biofiltration. The air normally humidifies before it enters the bed with a watering (spray) system, humidified chamber, bioscrubber, or biotrickling filter. Properly maintained, a natural organic packing media peat, vegetable mulch, bark, or wood chips may last for several years. However, engineered combined natural organic and synthetic component packing materials will generally last much longer, up to 10 years. A number of companies offer these types or proprietary packing materials and multiyear guarantees, not usually provided with a conventional compost or wood chip bed biofilter. For large volumes of air, a biofilter may be the only cost effective solution (http://www.biofilter.com/). There is no secondary pollution (unlike the case of incineration where additional CO_2_, CO, and NO gases are produced from burning fuel(s) and degradation products form additional biomass, carbon dioxide and water).

#### 10.1.2. Water Treatment

Trickling filters have been used to filter water for various end uses for almost two centuries. Biological treatment has been used in Europe to filter surface water for drinking purposes since the early 1900s and is now receiving more interest worldwide (http://www.biofilter.com/). Media irrigation water, although many systems recycle part of it to reduce operating costs, has a moderately high biochemical oxygen demand (BOD) and may require treatment before disposal. Biofilters are being utilized in Columbia falls, Montana at Plum Creed Timber Company's fibreboard plant (http://www.biofilter.com/).

 Biofiltration is one of the most effective water treatment technologies. Its application includes water filtration in farms, livestock operations, city municipal, industrial, and household applications. Some of the organisations which have supported the development or application biofiltration of water (http://www.biofilter.com/) over the past 14 years include the following:

- Prairie Farm Rehabilitation Administration (PFRA),
- The National Research Council (NRC),
- The Saskatchewan Research Council (SRC),
- Napier University (Scotland),
- Agriculture and Agro-food Canada.

Biofiltration is ideal for well, lake, pond, river, and dug out water. Biofilters remove the following substances from air and water: iron and iron bacteria, parasites, colour, cysts, manganese, pesticides, arsenic, lead, mercury, turbidity, dissolved organic carbon (dissolved organic material in water), tannins [26].

 A good number of research and practical work has been and is being carried out by Nigerian scientists and academics in the area of biofiltration of waste water. Bearing in mind that good water is a very essential commodity which is not readily available in most part of the country, it is therefore of great necessity to look at economic feasible ways to treat water for the benefit of the citizens. The sources of portable water for most Nigerian cities are government treated tap water and commercially treated drinking water as well as domestic water by water service private firms. Rural communities make do with water from ponds, streams, rivers, rain, and spring which are prone to contamination by water-borne diseases such as typhoid and diarrhoea which is common in those communities. Some of the applications of this important biotool in Nigeria are as follows.

1. Asamudo et al. [27] demonstrated the effectiveness of using the fungus *Phanerochaete chrysosporium *in the biofiltration of textile effluent, polycyclic aromatic hydrocarbons (PAH), and pulp and paper effluents. The microorganism was capable of producing extracellular enzymes such as manganese peroxidase, cellulases, and lignin peroxidases, in achieving total remediation of these effluents.
2. Ezeronye and Okerentugba [28] carried out a study to demonstrate the effectiveness of a yeast biofilter composed of a mixed culture of *Saccharomyces* spp., *Candida *spp., *Schizosaccharomyces* spp. and *Geotrichum candidum *in the treatment of fertilizer factory effluents and 98% treatment efficiency was achieved. The biochemical oxygen demand (BOD) of the effluent was reduced from a range of 1200–1400 to 135–404 mg/L. Besides, ammonia nitrogen (NH_3_-N) and nitrate-nitrogen (NO_3_-N) were reduced from 1000–10 mg/L and 100–17.6 mg/L, respectively.
3. Ogunlela and Ogunlana [29] developed a system using lava stones and oyster shells biofilter substrates for the oxidation of ammonia in a recirculatory aquaculture system. The effluent was treated using the biofilter, and chemical analyses were carried out once a week for four consecutive weeks. The results at the end of the fourth week indicated that the ammonia and nitrite concentrations were 0.0374 mg/L and 0.292 mg/L, respectively, which were below the permissible limits of 0.05 mg/L and 0.3 mg/L for ammonia and nitrite, respectively.

One of the most recent innovations in the use of this biotool in Nigeria was by Rabah et al. [30]. Their work describes the use of yeast biofilters in the treatment of abattoir waste water. Thus, Nono (locally fermented milk product) and Kunun-zaki (a refreshing drink made from millet) samples were obtained at the minimarket of the main campus of the Usmanu Danfodiyo University, Sokoto, Nigeria, in sterile sample bottles and transported in an icebox to the laboratory for the isolation of yeasts. Wastewater was collected from an abattoir in Sokoto, Nigeria, using sterile two litre capacity sample bottles and transported in an icebox to the laboratory. The wastewater was collected from three points in the abattoir: at the point where the wastewater leaves the slaughter hall (Point A, PA), midway through the drainage channel (Point B, PB), and the point where the wastewater drained to the surrounding soil (Point C, PC). A total of three samples were collected from each point at different times.

The biofilter was constructed using Perspex glass with a length of 18.0 cm, width of 10.8 cm, and a depth of 10.5 cm. The filter has upper and lower compartments separated by a perforated partition made up of the same Perspex glass. It also has a tap for the collection of filtered wastewater. Potato peels were ground to smaller particles, wetted, and placed on the perforated partition. The yeast biomass was inoculated on the peels and left for one week at ambient laboratory temperature (28 ± 2°C) to allow the cells to grow. Then the abattoir wastewater was introduced into the filter bed and left to stand for a minimum period of 14 days. The filtered wastewater was collected from the lower chamber of the filter through a tap fitted to the chamber. 

The yeast species isolated from the Nono and Kunun-zaki and identified for use as biofilters in the biofiltration process were identified as *Candida krusei, Candida morbosa, Torulopsis dattila, Torulopsis glabrata, *and *Saccharomyces chevalieri.* Also the results of the physicochemical qualities of the abattoir wastewater before and after biofiltration process from the three sampling points (PA, PB, and PC) revealed that there was a considerable reduction in pH, nitrate (NO_3_), dissolved oxygen (DO), biochemical oxygen demand (BOD), and chemical oxygen demand (COD) after the biofiltration of the wastewater collected from the three sampling points. It was also observed that the concentrations of other compounds in the wastewater varied with the sampling points probably due to contamination from human activities in the abattoir such as dumping of cow dung and pieces of bones in the wastewater channels. According to Rabah et al. [30], the results generally indicated that the yeast biofilter was fairly effective in the bioremediation process. The biofilter had a percentage efficiency of 42.5%.

### 10.2. Biomining

 Bacteria leaching is now used throughout the world as an additional technique for extracting metals from ores. Metals which can be extracted in this way include copper, uranium, cobalt, lead, nickel, and gold [31].

 Biomining is a generic term [32] that describes the processing of metal-containing ores and concentrates of metal containing ores using microbiological technology. Biomining has application as an alternative to more traditional physical-chemical methods of mineral processing. Commercial practices of biomining can be broadly categorized in two, namely, mineral biooxidation and bioleaching. Both processes use naturally occurring microorganisms to extract metals from sulphide bearing minerals. Minerals biooxidation refers to the process when it is applied to enhance the extraction of gold and silver, whereas bioleaching usually refers to the extraction of base metals, such as Zinc, Copper, and Nickel.

 Collectively, minerals biooxidation and bioleaching are commercially proven, biohydrometallurgical or biomining processes that are economic alternatives to smelting, roasting, and pressure oxidation to treat base and precious metals associated with sulphide minerals [32].

 Metals are essential physical components of the ecosystem, whose biologically available concentrations depend primarily on geological and biological processes [33]. Elevated levels of metals at specific sites can create a significant environmental and health problem when the release of metals through geological processes of decomposition and anthropogenic processes far exceeds that of natural processes of metal cycling. Metal contamination of both aqueous and terrestrial environments is of great concern, due to the toxicity and persistence of metals in the ecosystem and their threat to animal and human health [34]. Bacteria play an important role in the geochemical cycle of metals in the environment, and their capabilities and mechanisms in transforming toxic metals are of significant interest in the environmental remediation of contaminated sites. Microorganisms [34] colonize and shape the Earth in many ways, and their ability to adsorb and transform metals can shade light on solving pollution problems and proposing solutions in the clean up of contaminated site.

#### 10.2.1. Extraction Role of Microbes in Biomining

 Although many undiscovered microbial communities are involved in biomining, some of the popular and discovered bacteria responsible are: *Leptospirillum ferrooxidans*, *Acidithiobacillus thiooxidans,* and *Acidithiobacillus ferrooxidans* [31]. There is a good understanding of the exact role of microbes in biomining, thanks to today's sophisticated instrumentation that can examine materials at the atomic level. Given the fact that many microbes float freely in the solution around the minerals, many microbes attach to the mineral particles forming a biofilm [31]. The microbes, whether they are freely floating or whether they are in the biofilm, continuously devour their food sources—iron (chemically represented as Fe^2+^) and sulphur. The product of the microbial conversion of iron is “ferric iron,” chemically represented as “Fe^3+^”. According to Brierley [32], ferric iron is a powerful oxidizing agent, corroding metal sulphide minerals (e.g., pyrite arsenopyrite, chalcocite, and sphalerite) and degrading them into dissolved melts, such as copper, zinc, and more iron, the latter being the food source for the microbes. The sulphide portion of the mineral is converted by the microbes to sulphuric acid.

 Uranium occurs in oxidation states ranging from U (III) to U (VI), with the most stable species, U (VI) and U (IV), existing in the environment [34]. U (VI) is predominant in the oxic surface waters, and UO_2_^2+^ (uranyl) always forms stable, soluble complexes with ligands such as carbonate, phosphate, and humic substances [34]. In natural waters the solubility of U (VI) usually increases several orders of magnitude at higher pH values, due to complexation with carbonate or bicarbonate. By contrast, U (IV) is commonly found in the anoxic conditions and is present primarily as an insoluble uranite (UO_2_). Therefore, reduction of the soluble uranyl to the insoluble uranite seems to be an effective means to immobilize uranium in the anoxic environment to decrease the potential release of the mobile species [34].

 More research interests in the bioreduction of U (VI) are demonstrated in the dissimilatory metal-reducing bacteria (DMRB) under anaerobic conditions [34]. Lovley et al. [35] first demonstrated the occurrence of dissimulatory U (VI) reduction by the Fe (III) reducing bacteria *Geobacter metallireducens* and *Alteromonas putrifaciens* (later, *Shewanella putrefacians*), which could conserve energy for anaerobic growth via the reduction of U (VI). Soluble U (VI) is more readily reduced to U (IV) by *G. metallireducens* and other Fe (III) reducing microorganisms than are insoluble Fe (III) oxides, and once produced, U (IV) can be reoxidized to U (VI) with the reduction of Fe (III) to Fe (II) [36].

#### 10.2.2. Microbial Gold Mining

 In some precious-metal deposits gold [32] occurs as micrometer-sized particles that are occluded, or locked, within sulphide minerals, principally pyrite (an iron sulphide mineral) and arsenopyrite (an arsenic containing iron sulphide mineral). According to Brierley [32], to effectively recover the precious metals, the sulfides must be degraded (oxidized) to expose the precious metals. Once the sulphides are sufficiently degraded to expose the gold and silver, a dilute solution of cyanide is used to dissolve the precious metals. If the occluded gold and silver [32] are not exposed by breaking down the sulphide minerals, the cyanide cannot help in the release of the metals and recovery will be low. The ferric iron that is produced by the microorganism is the chemical agent that breaks down (oxidizes) the sulfide mineral. The microorganisms can be thought of as the manufacturing facility for producing the ferric iron. Microorganisms in the ore are destroyed by lime. Cyanide leaching can be accomplished in another heap or the oxidized and lime-conditioned ore can be ground and cyanide leached in a mill. The residue slurry is rinsed with fresh water, neutralized with lime, subjected to solid/liquid separation, and the solid residue is cyanide leaching to extract the gold. Gold recoveries are in the 95–98% range.

 Advantages of biomining [32] using organisms include the following.

1. Biomining microorganisms do not need to be genetically modified; they are used in their naturally occurring form.
2. Unlike humans, animals, and plants, microorganisms reproduce by doubling; that is, when there is abundant food (iron and sulfur) for biomining microbes and optimal conditions (sufficient oxygen, carbon dioxide and a sulfuric acid environment), a microbe will simply divide. Thus, in heap of minerals biooxidation for pretreating gold ores, there are about one million microbes per gram of ore.
3. High altitudes have no effect on the biomining microorganisms. However, additional air must be supplied to give the organisms an optimal performance.
4. Biomining using microorganisms does not produce dangerous waste products. Base metals, for example, zinc and copper are recycled and neutralized with lime/limestone.
5. The biomining microbes cannot escape from the heap or bioreactor to cause environmental problems. These microbes exist in the environment only where conditions are suitable (i.e., sources of iron and sulfur are oxidized, air and a sulfuric acid environment).

Biomining as a biotool has not been explored in Nigeria. Though Nigeria has many solid minerals in different states of the country, some of the minerals are tin (found in Plateau, Nassarawa, Kaduna, Bauchi and Gombe states), gold (found in Oyo, Osun and Ondo states), copper (Edo and Benue states), tantalite (Gombe, Plateau, Kaduna, and Nasarawa states), and uranium (Bauchi state) among others. The procedures, equipments, nonawareness/interest by government as well as competition with the physicochemical methods of extraction of these minerals are the greatest limitation in the exploitation and usage of this biotool in mining of minerals from their ores. Providing information to Nigeria scientists, ministries and government agencies is the solution to this limitation. Thus, the essence of the suggestion here is to create awareness in this regard.

### 10.3. Biomonitoring

 In a broad sense, biological monitoring involve any component that makes use of living organisms, whole or part as well as biological systems to detect any harmful, toxic, or deleterious change in the environment. There are various components employed in biomonitoring of contaminants in the environment. They include biomarkers (biological markers), biosensors, and many others.

 Biomonitoring or biological monitoring is a promising, reliable means of quantifying the negative effect of an environmental contaminant.

**Biological Markers**

A biomarker is an organism or part of it, which is used in soliciting the possible harmful effect of a pollutant on the environment or the biota [37]. Biological markers (biomarkers) are measurement in any biological specimen that will elucidate the relationship between exposure and effect such that adverse effects could be prevented [38]. The use of chlorophyll production in *Zea mays* to estimate deleterious effect of crude oil contaminants on soils is a typical plant biomarker of crude oil pollution [11]. When a contaminant interacts with an organism, substances like enzymes are generated as a response. Thus, measuring such substances in fluids and tissue can provide an indication or “marker” of contaminant exposure and biological effects resulting from the exposure. The term biomarker includes any such measurement that indicates an interaction between an environmental hazard and biological system [39]. It should be instituted whenever a waste discharge has a possible significant harm on the receiving ecosystem. It is preferred to chemical monitoring because the latter does not take into account factors of biological significance such as combined effects of the contaminants on DNA, protein, or membrane. Onwurah et al. [37] stated that some of the advantages of biomonitoring include the provision of natural integrating functions in dynamic media such as water and air, possible bioaccumulation of pollutant from 10^3^ to 10^6^ over the ambient value, and/or providing early warning signal to the human population over an impending danger due to a toxic substance. Microorganisms can be used as an indicator organism for toxicity assay or in risk assessment. Tests performed with bacteria are considered to be most reproducible, sensitive, simple, economic, and rapid [40] (Table 3).

### 10.4. Biosensor

**Figure 4:**
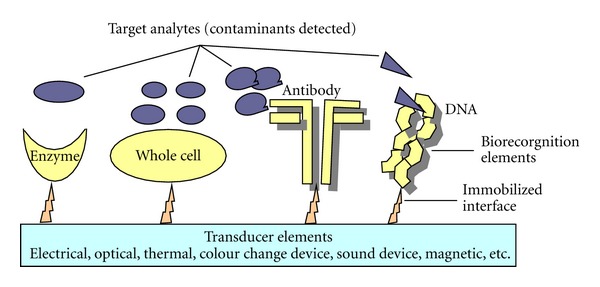
Anatomy of a Biosensor. The interaction between the target analyte and the biorecognition element creates a signalling event detectable by the interfaced transducer element. Modified from source: Ripp et al. [24].

 A biosensor is an analytical device consisting of a biocatalyst (enzyme, cell, or tissue) and a transducer, which can convert a biological or biochemical signal or response into a quantifiable electrical signal [46]. A biosensor could be divided into two component analytical devices comprising of a biological recognition element that outputs a measurable signal to an interfaced transducer [24]. Biorecognition typically relies on enzymes, whole cells, antibodies, or nucleic acids, whereas signal transduction exploits electrochemical (amperometric, chronoamperometric, potentiometric, field-effect transistors, conductometric, capacitative), optical (absorbance, reflectance, luminescence, chemiluminescence, bioluminescence, fluorescence, refractive index, light scattering), piezoelectric (mass sensitive quartz crystal microbalance), magnetic, or thermal (thermistor, pyroelectric) interfaces [24]. The biocatalyst component of most biosensors is immobilized on to a membrane or within a gel, such that the biocatalyst is held in intimate contact with the transducer and may be reused. Biosensors are already of major commercial importance, and their significance is likely to increase as the technology develops [46]. Biosensors are still emerging biotechnology for the future in environmental biomonitoring since they have specific limitations. Biosensors on a general sense are often employed for continuous monitoring of environmental contamination or as bioremediation process monitoring and biocontrol tools to provide informational data on what contaminants are present, where they are located, and a very sensitive and accurate evaluation of their concentrations in terms of bioavailability. Ripp et al. [24] explained that bioavailability measurements are central to environmental monitoring as well as risk assessment because they indicate the biological effect of the chemical, whether toxic, cytotoxic, genotoxic, mutagenic, carcinogenic, or endocrine disrupting, rather than mere chemical presence as is achieved with analytical instruments. As the name suggests they are biological instruments that detest and signal the presence of harmful contaminants in the environment. There are different types based on the biological components on which their sensitivities are based (Figure 4). Some of them, though not exhaustive are the following.

#### 10.4.1. Enzyme-Based Biosensors

**Figure 5:**
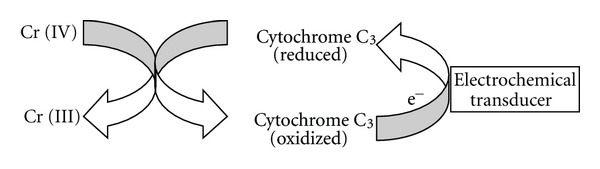
Enzymatic biosensor using cytochrome C_3_ as the recognition element. Upon exposure to chromate [Cr (VI)], electrode-immobilized cytochrome C_3_  reduces Cr (VI) to Cr (III). The current produced by the electrochemical regeneration of reduced cytochrome C_3_ is proportional to the amount of oxidized cytochrome C_3_ and, therefore, the Cr (IV) concentration. Ripp et al. [24].

 Leyland Clark in the 1960s used an enzyme biosensor which consists of glucose oxidase enzyme immobilized on an oxygen electrode for blood glucose sensing. This historical application of enzyme-based biosensor has found a world-wide lucrative application in medical diagnosis. Nevertheless, enzyme-based biosensor gradually gained application in environmental monitoring. According to Ripp et al. [24], enzymes act as organic catalysts, mediating the reactions that convert substrate into product. Since enzymes are highly specific for their particular substrate, the simplest and most selective enzyme-based biosensors merely monitor enzyme activity directly in the presence of the substrate. A novel example of biosensors of enzyme origin which has found application in the environment is the sulfur/sulfate-reducing bacterial cytochrome C_3_ reductases that reduce heavy metals. Michel et al. [47] immobilized cytochrome C_3 _on a glassy carbon electrode and monitored its redox activity amperometrically in the presence of chromate [Cr (IV)] with fair sensitivity (lower detection limit of 0.2 mg/L) and rapid response (several minutes) (Figure 5).

When tested under simulated groundwater conditions, the biosensors reacted with several other metal species, albeit at lower sensitivities, and were affected by environmental variables such as pH, temperature, and dissolved oxygen. Similarly operated enzyme-based biosensors for ground water contaminant perchlorate using perchlorate reductase as the reduction enzyme (detection limit of 10 **μ*g*/L) [48], organophosphate pesticides using parathion hydrolase or organophosphorus hydrolase as recognition enzymes (detection down to low **μ**M concentrations) [49], and environmental estrogens using tyrosinase as the recorgnition enzyme (detection down to 1 **μ**M) [50] have also been designed.

Another type of enzyme biosensor relies on enzyme activation upon interaction with the target of interest. Heavy metals, for example, in the form of cofactors-inorganic ions that binds to and activate the enzyme can be detected based on this integral association. Metalloenzymes such as alkaline phosphatase, ascorbate oxidase, glutamine synthetase, and carbonic anhydrase require association of a metal ion cofactor with their active sites for catalytic activity and can thus be used as recognition element for heavy metal [24]. Alkaline phosphatase, for example, can be applied in this regard as a biosensor for zinc [Zn (II)] or ascorbate oxidase for biosensing copper (II) with detection limits down to very low part-per-billion levels [51]. Various immobilization techniques are adopted in the attachment of the enzyme to the transducing element [52]; they include adsorption, covalent attachment, entrapment in polymeric matrices such as sol-gels or Langmuir-Blodgett films, or direct cross-linking using polymer networks or antibody/enzyme conjugates. Immobilization provides the biosensor longevity and with recent integration of redox active carbon-based nanomaterials (nanofibers, nanotubes, nanowires, and nanoparticles) as transducers and their unique ability to interact with biological material, a promising advancement in enzyme biosensor design and sensitivity is in sight.

 Optical transducers (absorption, reflectance, luminescence, chemiluminescence, evanescent wave, surface plasma resonance) are also commonly employed in enzyme-based biosensor [24]. This can be as simple as optically registering a pH change using a pH reactive dye; for example, bromocresol purple can be immobilized with an acetylcholinesterase-based biosensor to monitor pH changes related to this enzyme's activity upon exposure to pesticides. Acetylcholinesterase hydrolysis releases protons (H^+^), resulting in a decrease in pH, which in turn instigates a decrease in the absorption spectra of bromocresol purple [24].

 Andreou et al. [53] incorporated such a biosensor successfully on the distal end of a fibre optic cable for facile interrogation of water samples for pesticide residue. A great application of optical biosensor is in the Luminol, widely used as an electrochemiluminescent indicator. It reacts with the acetylcholinesterase/choline oxidase hydrogen peroxide by-product to yield luminescent light signals that have also been used to quantify pesticide concentrations.

#### 10.4.2. Antibody-Based Biosensors (Immunosensors)

 These types of biosensors make use of antibodies as recognition elements (immunosensors). They are used widely as environmental monitors because antibodies are highly specific, versatile, and bind stably and strongly to target analytes (antigens) [24]. Antibodies can be highly effective detectors for environmental contaminants, and advancements in techniques such as phage display for the preparation and selection of recombinant antibodies with novel binding properties assures their continued environmental application. Perhaps the best introduction to antibody-based biosensing is the Automated Water Analyzer Computer Supported System (AWACSS) environmental monitoring system developed for remote, unattended, and continuous detection of organic pollutants for water quality control [54]. AWACSS uses an optical evanescent wave transducer and fluorescently labelled polyclonal antibodies for multiplexed detection of targeted groups of contaminants, including endocrine disruptors, pesticides, industrial chemicals, pharmaceuticals, and other priority pollutants, without requisite sample preprocessing. Antibody binding to a target sample analyte occurs in a short 5-minute preincubation step, followed by microfluidic pumping of the sample over the transducer element, which consists of an optical waveguide chip impregnated with 32 separate wells of immobilized antigen derivatives [24]. As the antibody/analyte complexes flow through these wells, only antibodies with free binding sites can attach to the well surface (in what is referred to as a binding inhibition assay). Thus, antibodies with both of their binding sites bound with analyte will not attach to the surface and will pass through the detector. A semiconductor laser then excites the fluorophore label of bound antibodies, allowing for their quantification, with high fluorescence signals indicating high analyte concentrations. A fibre optic array tied to each well permits separation and identification of signals by the well, thereby yielding a simultaneous measurement of up to 32 different sample contaminants. The instrument has been used for groundwater, wastewater, surface water, and sediment sample testing with detection limits for most analytes in the ng/L range within assay times of approximately 18 minutes [24]. Another design by Glass et al. [55], similar to the above but less refined benchtop flow-through immunosensor (KinExA) was demonstrated to detect analytes successively based on a replaceable flow cell containing fluorescently labeled antibody. Their time of assay was approximately 26 minutes, with detection limits at picomolar concentrations.

 Although not as elaborate as the AWACSS, a multitude of other antibody-based biosensors have been applied as environmental monitors, traditionally serving as biosensors for pesticides and herbicides, but their target analytes have broadened considerably over the past several years to include heavy metals, polycyclic aromatic hydrocarbons (PAHs), polychlorinated biphenyls (PCBs), explosives (TNT and RDX), phenols, toxins such as microcystin, pharmaceutical compounds, and endocrine disruptors [56].

#### 10.4.3. DNA-Based Biosensors

 The principle underlying the DNA-based biosensor is the ability of a transducer to monitor a change in the nucleic acid's structure occurring after exposure to a target chemical. These structural changes are brought on either by the mutagenic nature of the chemical, resulting in mutations, intercalations, and/or strand breaks, or by the chemical's ability to covalently or noncovalently attach to the nucleic acid [24]. Immobilizing the nucleic acid as a recognition layer on the transducer surface forms the biosensor, and detection of the chemically induced nucleic acid conformational change is then typically achieved electrochemically (i.e., a change in the current) or less so through optical or other means [57].

 Nucleic acid biosensors are generally nonselective and provide an overall indication of a potentially harmful (genotoxic, carcinogenic, cytotoxic) chemical or chemical mix in the test environment and, depending on the biosensor format, an estimate of concentration. Bagni et al. [58] illustrated a conventional DNA biosensor which was used to screen soil samples for genotoxic compounds, using benzene, naphthalene, and anthracene derivatives as model targets. Double-stranded DNA was immobilized on a single-use disposable screen-printed electrochemical cell operating off a handheld battery-powered potentiostat [59]. A 10 **μ**L drop of a preprocessed and preextracted contaminated soil sample was placed onto the working electrode for 2 minutes, and resulting electrochemical scans, based on the chemical's propensity to oxidize DNA guanine residues, were measured. The magnitude of these “guanine peaks” in relation to a reference electrode was linearly related to their concentration in solution (i.e., the higher the concentration of the target chemical, the more the damage imposed on the DNA, and the lower the electrochemical measurement of the oxidation signal). In a very discrete application of this DNA biosensor, the authors also applied it to the detection of this DNA biosensor and also to the detection of PAHs in fish bile, using the accumulation of PAH compounds in live fish to monitor for water contamination events [60]. 

Nucleic acid can be manipulated similarly to create target specific aptamers using a process called SELEX (systematic evolution of ligands by exponential enrichment) [24]. By iteratively incubating nucleic acid with the desired target, one can select for oligonucleotide sequences (or aptamers) with the greatest affinity for the target. Kim et al. [61] used SELEX to create an aptamer specific for 17 *β*-estradiol and used it in an electrochemical biosensor (or aptasensor) to achieve detection of this important endocrine disruptor at levels as low as 0.1 nM. Predominant aptasensor development and application is in the clinical fields, but it is slowly and inevitably encroaching upon environmental sensing. An aptasensor for the cyanobacterial toxin microcystin (lower dertection limit of 50 **μ**g*/*mL) [62] and another for zinc based on fluorophore beacon (lower detection limit of 5 **μ**M) [63] have been reported.

 Hydrazine and aromatic amine compounds in fresh and groundwater, hydroxyl radicals in uranium mine drainage waters, herbicides such as atrazine, general toxicity events in wastewater, industrially contaminated soils, and various other environmental sources have all been screened using DNA biosensors [24].

 Metals are also relevant detection targets, due to their various affinities for nucleic acid. Lead, Cadmium, Nickel, Arsenic, Copper, Iron, Chromium, and others have been detected through DNA biosensing, incorporating both single- and double-stranded DNA as the sensing element, but again, nonselectivity [24]. Selectivity, though, has been demonstrated by several groups using deoxyribozymes (DNAzymes) or ribozymes (RNAzymes). These engineered catalytic oligonucleotides can mediate nucleic acid cleavages or ligation, phosphorylation, or other reactions. For example, DNAzyme biosensor for lead uses a single-stranded DNAzyme absorbed to a gold electrode [64]. The DNAzyme incorporates a methylene blue tag at concentrations as low as 62 ppb; the DNAzyme strand is cleaved, allowing the methylene blue tag to approach the transducer and transfer electrons, thereby instigating an electrochemical signal [24]. However, the rapidity (only a few minutes to detect but sample processing is often necessary), sensitivity (typically down to low part-per-billion levels), ease of use, and cost-effectiveness screen environmental sites for toxic chemical intrusions or monitoring operational endpoints of bioremediation efforts. A calorimetric DNAzyme-based biosensor for lead has also been demonstrated [65].

#### 10.4.4. Biomimetics, BioMEMs, and Other Emerging Biosensor Technologies

 The future of biosensors is clearly in the emergent technologies of Biomimetics and BioMEMs. BioMEMs (biological microelectromechanical systems) are an assortment of biomicro, bioanotechnological, and microfluidic interfaces that form lab-on-a-chip, biochip, or micrototal analysis system (**μ** TAS) biosensors [24]. Their objectives are toward miniaturization, portability, redundancy, and a reduction in sample size, time of response, and cost. The majority of these biosensors serves biomedical rather than environmental causes, but they are slowly and inevitably being adapted for the environmental monitoring community. BioMEMs most often utilize optical transducers interfaced with enzyme, whole-cell, antibody, or nucleic acid-type receptors. Several recent examples should illustrate their various design and performance characteristics. Yakovleva et al. [66] developed a microfluidic immunosensor flow cell for the detection of atrazine in surface water. Chemiluminescently labelled antibodies directed against atrazine were combined with artificially contaminated river water and microfluidically pumped at 40 to 50 **μ**L*/*minute through a 42-channel 13 mm × 3 mm silicon microchip containing a functionalized antibody affinity-capture surface [24]. Upon antibody/atrazine capture, a luminal substrate was added to mediate the chemiluminescent reaction which was monitored with a photomultiplier tube (PMT) suspended above the microchip. Islam et al. [67] have further improved this sensing strategy by essentially integrating the PMT directly on the microchip flow cell to create truly miniaturized biosensor referred to as a BBIC (bioluminescent bioreporter integrated circuit). This 1.5 mm × 1.5 mm CMOS microluminometer was designed to capture and process bioluminescent signals emanating from immobilized whole cell bioluminescent bioreporter bacteria [24]. The BBIC converts the bioluminescently derived photodiode current into a digital signal, the frequency of which is proportional to the concentration of pollutant to which the bioreporter has been exposed. In water artificially contaminated with salicylate as a model pollutant, the flow-through BBIC responded with 30 minutes to part-per-billion concentration [24].

 BioMEMs also include microcantilever-based biosensors that translate a molecular recognition event into nanomechanical motion that is measured by induced bending in a microfabricated cantilever similar on a macroscale to identifying a person on a diving board based on the deflection of the diving board by their weight. Optical or piezoresistive transducers usually measure microcantilever deflections at nanometer-to-subnanometer ranges of motion, and due to their small size, several microcantilevers can be accommodated per transducer for multianalyte sensing. Alvarez et al. [68] immobilized antibodies to the pesticide DDT on a microcantilever and demonstrated real-time detection at nanomolar concentrations.

 Biomimetics mimic (imitate) the attributes of naturally occurring biological materials to synthetically recreate or enhance their properties [24]. Molecularly imprinted polymers (MIPs) are one of the typical examples of Biomimetics application in biosensors which can deliver more robust, stable, and target-specific receptors. MIPs are essentially created by mixing the target analyte (or template) with a monomer. Resulting MIPs then serve as analyte-specific synthetic receptors (or artificial antibodies or enzymes) that can be associated with transducers to form sensors. Dickert et al. [69] synthesized MIP receptors for various PAH constituents, optically interrogated them with a fluorescent sensor, and demonstrated detection of individual PAHs such as pyrene down to ng/L concentrations in artificially contaminated drinking water. More recently, Xie et al. [70] molecularly imprinted the explosive 2,4,6-trinitrotoluene (TNT) onto the walls of silica nanotubes, thus implying a great future for MIP nanosensors which have faster response time and great sensitivity. Other environmentally relevant MIP sensors have been designed for various herbicides/pesticides (2,4-D, atrazine, phenylureas, CAT, DDT), aquatic toxins such as microcystin and demoic acid, and various heavy metals, with incorporation into a variety of optical, electrochemical, or piezoelectric transducer element [71].

 Biosensors based on the use of whole animals or their organs represent a very unique mode of sensing. Insect antennas, for example, are covered with highly sensitive and naturally tuned receptors called sensilla that respond to chemical, physical, and mechanical signals via electrical nerve impulses. By immobilizing the antenna or even the entire insect on a transducer and measuring these induced electrical impulses (or electroantennograms), a biosensor materializes [24]. A multianalyte biosensor can be formed by adhering antennas from several different insects. The current targets for such biosensors are odourants such as those related to smoke (guaiacol and 1-octen) for early-warning fire detection or volatiles emanating from diseased plants, with detection limits in the part-per-billion range. Their parallel applications for sensing volatiles associated with environmental contaminants and even non-odour-related compounds are a potential future prospect [24]. Imagine a chip sensitive to a particular pollutant analyte attached to a fish, insects, or invertebrate and the organism released into the environment while the sensor is monitored with a computer. The possibility is as much as the imagination can go!

### 10.5. Biomass Fuel

 Biomass is any plant or animal matter used to produce energy. Many plants and plant-derived materials can be used for energy production; the most common is wood. Other sources include food crops, grasses, agricultural residues, manure, and methane from landfills [72]. The main driving forces for adoption of biomass fuel and its encouragement is mainly due to the more efficient bioprocesses and bioproducts which are cost savings and improved product quality/performance. Environmental consideration of the quick degradation of by-products of biomass fuel is a major consideration in the development of this biotool.

 The state of Texas in the United States of America is known to be an agricultural state that has adopted biomass energy production. Crops used to produce biomass energy include cotton, corn, and some soybeans—all grown in Texas [73]. In the US, the primary biomass fuels are wood, biofuels, and various waste products. Biofuels include alcohols, synfuel, and bio-diesel, a fuel made from grain and animal fats. Waste consists of municipal solid waste, landfill gas, agricultural by-products, and other material. Most biomass energy used in the US—65 percent—comes from wood [74]. Another 23 percent of biomass energy used comes from biofuels. while the remaining 12 percent comes from waste energy. Energy generated from biomass is the nation's largest source of renewable energy, accounting for 48 percent of the total in 2006. The US consumed 3,277 trillion British thermal units (Btu) of biomass energy in 2006. The next largest source of renewable energy is hydroelectric power, with 2,889 trillion Btu consumed in 2006 [75].

 While cattle manure has the most potential for power use, other forms of agricultural waste have significant possibilities, too. These include poultry litter, rice straw and husk, peanut shells, cotton gin trash, and corn stover. In fact, a recent report from the Houston Advanced Research Center estimated that Texas agricultural wastes have the potential to produce 418.9 megawatts of electricity, or enough to power over 250,000 homes, based on average Texas electricity use in 2006 [76].

 Plant biomass can be processed and converted by fermentation and other processes into chemicals, fuels and materials that are renewable and result in no net emissions of greenhouse gases. Also, energy, such as waste heat, can be used efficiently. This approach is called industrial ecology [77].

 In the US, ethanol made from corn currently accounts for the majority of biofuel consumption in the transportation sector. In the future, however, “lignocellulosic” biofuels made from crop residue, grasses, wood products, sorghum, “energy cane,” and agricultural waste are expected to supplement corn ethanol. These are commonly referred to as “cellulosic.” Public and private funding for new research in cellullosic fuels is increasing. Corn ethanol requires significant amounts of fertilizers, pesticides, energy and water to grow, cellulosic biofuel production promises to be much more efficient. The production of cellulosic ethanol and other biofuels is expected to give significant increase in yield of fertilizers, pesticides and energy production. Cellulosic biofuel production promises to be much more efficient in economic usage of agricultural products. 

 Biologically derived products (bioproducts) are generally less toxic and less persistent than their petrochemical counterparts. Group of companies can mimic the cooperative action of organisms in natural ecosystems by clustering around the processing of a feedstock such as a biomass so the by-product of one is the starting material for another. The ability to evolve bioprocesses and bioproduction systems allows for major improvements in both economic and environmental performance. This permits a manufacturing facility to increase its profitability and capacity while maintaining or even reducing its environmental footprint [77].

In Nigeria, lots of projects are ongoing in the area of biofuel. Dependence on crude oil as a major economic stay for the country has also had its toll on other sectors such as agriculture. Despite this, Nigeria is yet to develop the refining sector of the crude oil exploration. Nigeria currently imports refined petroleum, diesel, ethanol, and kerosene from countries around the world. This has not adversely affected the price of the commodity in the country. Its negative effect is slowly being realized. As a result, it is wise to quickly look at alternative sources of energy so as to avoid the short fall of overdependence on nonrenewable sources such as the petroleum industry so as to cushion the gradual but sure effect of this activity in the economy of the country. The government is also gradually looking at ways to refine the crude oil by revitalizing the refineries and making them functional. Despite all these measures, biofuel still holds the answer to the solution of the problems in the oil sector as long as the environment is concerned. Also the future portends that better industrial management practices will preferentially make use of agro-based sources in all production process so as to put the environment first in all production practices. Two of such ongoing research in this biotechnological tool process involve: Alkali-catalysed Laboratory Production and Testing of Biodiesel Fuel from Nigerian Palm Kernel Oil by Alamu et al. [78] and BioDiesel Nigeria (BDN), a company that will provide jobs for Nigerians by farming Jatropha seeds. The company had proposed on providing employments for those living in poverty in the cities by relocating them to Jatropha farms in the north. There they will be trained to farm Jatropha trees and be paid a decent wage. Tables 4 and 5 show recorded extent of Biofuel efforts in Nigeria.

Highina et al. [80] stated that biofuel industry in Nigeria is still at its infancy, even though policy guidelines are available at the NNPC for the development of the industry, few most ground breaking achievements have been made. In 2007 the Kaduna State Government of Nigeria set up a pilot plant for bioethanol production to demonstrate the technology and its viability using local design and materials. Ahmadu Bello University, Zaria, Kaduna State Nigeria, also established a pilot plant for biodiesel production Bugaje and Mohammed [81]. Other efforts elsewhere in the country have been limited to bench-scale production of bioethanol and biodiesel from a number of feedstock. There is need to move further from this and scale up for commercial production. Ethanol is produced from fermentation of sugars and biodiesel from the reaction of plant or animal oils with an alcohol, for which methanol is universally used in commercial application today [80].

### 10.6. Aquaculture Treatment/Management

 Catfish cultivation has assumed a commercial dimension world-wide. With the adoption of this agricultural wealth creation technique, and the uniqueness of its good protein content, it has been accepted as special delicacy among the wealthy especially in Nigeria. Most families in south-eastern and south-western Nigeria cultivate catfish on medium to commercial scale as a financial supplement for regular job. Most individual, have also engaged themselves as full-time catfish farmers in different level of this aquaculture business. Thus, some are into catfish feed production, breeding (reproduction) as well as total development and marketing of this agricultural product. Apart from the fact that there are various feeds available in the market for cultivation of catfish, various feed supplements from poultry waste to domestic food remnants as well as vegetables, nuts, fruit peels, and so forth are also integrated in the production of this fast growing aquaculture practice. In line with this encouraging agricultural venture is the attendant problem of effluent generation which can adversely affect the aesthetic environments.

 Catfish pond effluent quality varies from pond to pond and from season to season. Effluent quality is usually poorest (highest concentrations of solids, organic matter, total phosphorus, and total nitrogen) in the summer when fish feeding rates and water temperatures are highest. Catfish pond effluents generally have higher concentrations of nutrients and organic matter than natural stream waters but much lower concentrations than municipal, and industrial waste water. It appears that catfish pond effluents are most likely to exceed regulatory limits for suspended solids and total phosphorus. Other measured water qualities sometimes are used for treating agricultural, municipal and industrial waste water.

 According to SRAC [82] report, there are various biological techniques adopted, which are cost efficient and eco-friendly in the treatment of aquaculture effluents. Some of such aquaculture biotechniques include the following.

**Construction of Wetlands Adjacent to Ponds**

Wetlands are inexpensive to build and operate, and it also eliminates the need for chemical treatment of wastewater. They also contribute stability to local hydrologic processes and are excellent wild-life habitats.

The disadvantage of wetland for treating aquaculture pond wastes is the large amount of space necessary to provide an adequate hydraulic residence time. Therefore, it will probably be necessary to integrate wetland treatment of effluents with other pond effluent management procedures to reduce the area of wetland needed. For example, a wetland centrally located on a farm, or connected to an integrated drainage system, would save on construction costs and use land efficiently. Such a system would also allow a wetland to be used to treat the overflow coming from ponds after rainfall. Pond draining could be staged so that only one pond is being drained at a time, allowing one wetland to serve numerous ponds. Effluent from a constructed wetland could even be pumped back into ponds and reused if needed.

Wetlands act as biological filters to remove pollutants from water, and natural or constructed wetland exposes the solid, semisolid waste particles for fast degradation by microorganism. The water if properly channeled can be reused, and the dried solid component of the effluent when properly treated can be used as organic fertilizer in crop production [82].

#### 10.6.1. Treating Pond Effluents Using Grass Filter Strips

 Draining effluents over grass strips filters solids from animal waste. This system may be useful for filtering catfish pond effluent. Common and coastal Bermuda, dallies, and Bahia are recommended grasses for warm climates; fescue, reed canary, and rye grasses are recommended for cool climates. Grass filter strips are highly effective in reducing the concentrations of suspended solids, biochemical oxygen demand, and ammonia, but not efficient in removing algae.

 Concentrations of suspended solids, organic matter, and total nitrogen in catfish pond effluents were reduced by applying the effluent to well-established strips of either Bahia or Bermuda grass. This filtering technique was relatively easy and inexpensive and may have application if the filtered effluent is to be reused for fish production to conserve groundwater. It could also be used to treat effluent before discharging it to receiving water [82].

#### 10.6.2. Management Practices to Reduce the Impact of Aquaculture Effluents on the Environment

**(a) Conservative Water Management Practices**

This involves two major techniques, namely,

1. reusing water for Multiple Fish Crops
2. reducing overflow after rains by keeping pond water level below the pond drain.

**Reusing Water for Multiple Fish Crops**

The concentration of the substance in the effluent is dependent on the volume of water discharged as well as the mass of nutrients (organic matter) present in the ponds according to the aquacultural feeding history. Thus, reducing the concentration of potential pollutants in pond effluents is difficult, but it is relatively easy to control discharge volume. The most obvious procedure for reducing the volume of effluents from channels catfish ponds is to harvest the fish without draining the ponds. However, this practice works only if water quality does not deteriorate as the water is reused.

SRAC [82] report established that a comparison of annually drained and undrained catfish ponds showed little difference in water quality and no difference in fish production. Natural processes, such as nutrient uptake by bottom soils, microbial decomposition of organic matter, denitrification and sedimentation, continually remove potential pollutants from pond water. Operating ponds without draining makes better use of the waste assimilation capacity of ponds and saves significant amounts of water as well as reducing overall effluent volume.

**Reducing Overflow after Rains by Keeping Pond Water Level below the Pond Drain**

Seasonal changes in overflow volume affected the amount of waste discharged more than seasonal changes in effluent quality [82]. So, reducing overflow volume can have a dramatic impact on mass discharge of nutrients and organic matter from catfish ponds. By keeping the pond water level below the level of the drain, rainfall is captured rather than allowed to overflow and annual waste discharge is reduced by 50 to 100% depending on the weather. Specifically, waste discharges are normally greatest in winter when overflow volume are highest and not in summer when waste concentrations are highest. A study was conducted in which effluents from Georgia catfish ponds were used to determine the production of soybean irrigated with such effluents.

**(b) Using Effluents for Irrigation of Soybeans**

Although water discharged from aquaculture ponds is often viewed simply as a waste product, it still has value and its reuse may have multiple benefits. If ponds are located near terrestrial crops that require irrigation, pond discharge can be used for irrigation water. That use will reduce waste discharge and benefit the crop. The SRAC [82] report has it that the total nitrogen available for crops varied from 0.9 to 1.2 kg/ha from each centimeter of water applied. Assuming average irrigation is 30 cm, then available nitrogen ranged from 27 to 36 kg/ha, a significant portion of the nitrogen requirement of many agronomic crops. Although the average soybean yield was 3.6 metric tons/ha, double the average yield in Georgia, the increased yield was the result of irrigation alone and not the nutrients in the irrigation water. Although the nutrient content of pond effluents may be too low to affect crop production, effluent water not useful for catfish cultivation can find application for irrigation of crops and thus reduce discharge volume. Rice irrigation with effluents of aquaculture has also been suggested.

It is important to note that even though catfish farming is the major aquaculture, there are also other aqua cultural practices like cultivation (breeding) of crawfish, shrimps, and other fresh water organisms.

The summary of the SRAC [82] publication is an examination of the impact of aquaculture pond effluent on the environment and how the use of cost reduction and yet simple management practices can help to control pollution of the environment with aquaculture effluents. These simple management practices do not require extra expense or labour. It is advocated therefore that all aquaculturists should strive to reduce the impact of their activities on the environment by adhering to the following guidelines (aquaculture management practices).

- Use high-quality feeds and efficient feeding practices. Feeds are the origin of all pollutants in catfish pond effluents.
- Provide adequate aeration and circulation of pond water. Maintaining good dissolved oxygen levels enhances the appetite of fish and encourages good feeding conversion. Oxygen availability at bottom of ponds improves degradation of organic matter and reduces the amount of organic matter in effluent.
- Minimize water exchange. Routine water exchange is of questionable value as a water quality management procedure and greatly increases effluent volume.
- Operate ponds for several years without draining. Reusing water for multiple fish crops is one of the best methods of reducing waste discharge from ponds.
- Capture rainfall to reduce pond overflow also reduces the need for pumped water to maintain pond water levels.
- Allow solids to settle before discharging water. After sieving ponds partially drained for fish harvest, hold remaining water for 2 to 3 days to allow solids settle. Better still dose not discharge this last portion of water.
- Reuse water that is drained from ponds. Instead of draining ponds for fish harvest, water can be pumped to adjacent ponds and reused in the same or other ponds.
- Treat effluents by using constructed wetlands.
- Use effluents to irrigate terrestrial crops. Under certain conditions, the water discharged from ponds may have value as irrigation water for crops.

### 10.7. Biocatalysts

 By using well-established tools from metabolic engineering [83] and biochemistry [84], efforts have been made on engineering microbes to function as “designer biocatalysis,” in which certain desirable traits are brought together with the aim of optimizing the rate and specificity of biodegradation. Therefore, enzymes extracted from naturally occurring microorganisms, plant and animals can be used biologically to catalyse chemical reactions with high efficiency and specificity. Compared to conventional chemical processes, biocatalytic processes usually consume less energy, produce less waste, and use less organic solvents (that then require treatment and disposal) [77].

**Figure 6:**
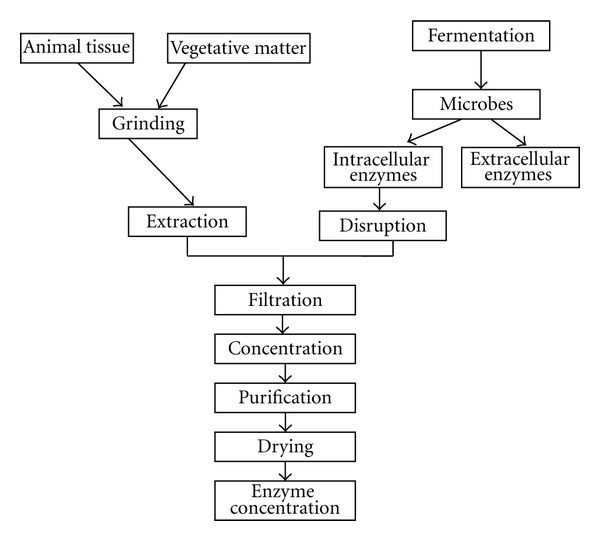
Steps in industrial fermentation (enzyme production). Source: [25].

 Microbial industrial production of enzyme involves a lot of aerobic steps within a submerged culture in a stirred tank reactor. The enzyme biochemistry is driven by transcription, translation, and molecular mass, number of polypeptide chains, isoelectric point, and degree of glycosylation, such as a saccharide's reaction with a hydroxyl or amino group to form a glycoside [20]. The selection of microbes as candidates for fermentation depends on process characteristics (such as viscosity or recoverability), legal approval of use, and the state of knowledge about the selected organism. Sugars comprise the principal feedstock (i.e., production process strictly biological) for microbial processes (carbon and energy sources) [20]. Feedstocks include molasses, unrefined sugar, and sulfite liquor from cellulose production plants, hydrolysates of wood and starch, or fruit juices, such as the grape juice used in wine making processes. Thus, these raw sources contain other compounds beside sugars. This can be beneficial, because vegetative materials invariably contain nitrogen, phosphorus, and potassium, important nutrients to maintain microbial growth and metabolism [20]. For the purer feed stocks, the nutrients are added to the reactor as inorganic compounds such as ammonium compounds, phosphate, and potassium chloride. Organic supplements include meal, fish meal, cotton seed, low-quality protein materials such as casein or its hydrolysates, millet, stillage, and corn steep liquor. In addition, these chemically complicated mixtures must contain micronutrients, that is trace elements and growth promoters, which are limiting factors. In general, the raw materials are dissolved or suspended in water, and then the medium is heated, filtered, and sterilized. For downstream processing (harvest, concentration, and purification) or for analytical assays during the process, additional pretreatment of the raw material can reduce unwanted side reactions [20] (Figure 6).

By imitating natural selection and evolution, the performance of naturally occurring enzymes can be improved. Enzymes can rapidly be “evolved” (this technique is called “molecular evolution”) through mutation or genetic engineering and selected using high-throughput screening to catalyse specific chemical reactions and to optimize their performance under certain conditions such as elevated temperature [77].

## 11. Nigeria Situation

In research institutions and universities, biocatalysis have been applied in laboratory-scale experiments and primary investigation of product synthesis. Most of the detergents in the country are incorporated with various enzymes. The most significant areas of application of biocatalysis as a biotechnological tool in Nigeria are in the areas of yoghurt production by many local industries, confectionaries and bread production industries, local fermented special seasonings (Ogiri and Okpei) for soup making in the eastern part of Nigeria, produced by boiling melon seed, castor oil seeds and exposing them to microorganisms in the environment for fermentation followed by milling and packaging for consumption. Others involve fermentation of cassava for food and brewing of alcoholic drinks by pilot commercial industrial production in organizations such as Nigerian Breweries, Nigerian Distilleries, Guinness Nigeria Plc, and many other brewing industries which spread across various states in the country (Table 6).

Enzyme biotechnologies can be visualized as sets of biological reactions occurring at various scales in the environment. The activities of enzyme in reactions can lead to desirable results, such as the chemical transformation and ultimate degradation of toxic substances into harmless compounds. Biological reactions may also lead to undesirable results, such as the introduction of genetically modified organisms to an ecosystem or the generation of toxic chemicals. Here enzymes are modified to do the clearing process of these toxins.

## 12. Conclusion

 At the backdrop of the need to meet certain challenges that affect development, thus the much needed change to bring about these developments informed the decision by the United Nation to elaborate on key agenda which most nations are expected to adhere to in order to achieve certain goals known as Millennium Development Goals (MDGs). The attainment of these goals, aiming at ensuring that participating countries, provide basic good things of life for their citizens.

 The United Nations (UN) Secretary General's Special Adviser on the MDGs, Jeffrey D. Sachs, visited Nigeria recently for assessment of progress in indicators of whether Nigeria is on the part of attaining these (MDGs) and he gave his verdict. In his word as reported by Anuforo [94] “One would say Nigeria is on the path, but not well on the path. The direction is positive. The institutional innovation is exciting. But the quantitative achievement is not sufficient. So, there really need to be acceleration between 2010 and 2015.”

 On her part, the Senior Special Assistant to President Goodluck Jonathan on the MDGs, Mrs. Amina Az-Zubair, also gave perspective to what the Nigerian Government is doing to attain the 2007 to 2016 objectives. According to Agbaegbu [95], Az-Zubair said that the overarching objective of the countdown towards Nigerians achievement of the MDGs by 2015, on to safe water and sanitation have not improved significantly, and other environmental challenges such as erosion, coastal flooding, and climate change are growing. The MDG office reported, however, that the proportion of the population with access to safe drinking water dropped from 54 percent in 1990 to 49 percent in 2007. The proportion of the population with access to basic sanitation is said to have risen from 39 to 43 percent in the recent period. “With better implementation of the various policy frameworks and plans for water, sanitation, environment and slum upgrading, Nigeria will be on track to achieve this goal,” the MDG office report said. Even so, it noted that “planning and maintenance of water supply at local level is weak. Environmental pressures range from desertification in the North to flooding, rubbish heaps and soil erosion in the coastal and Niger Delta regions, requiring a nationally coherent but localized approach.”

 Enduring sustenance of the environment must come from using the natural methods to remove the synthetic or deleterious activities of man in the environment. When a forest, for example, loses its trees it takes some time to regenerate but when it does it retains it natural beauty and improve the quality of other biota within it. When a lake is contaminated with pollutants, prevention of more pollution and exploitation of gases (aeration), encouragement of microbial activities restores the health of the lake. Thus, biotechnological tools are bioenvironmental technological practices aimed at encouraging without compromise with any other technology no matter how fast and efficient those other technologies could be in restoring the environment in such a way that is closer to nature if not totally natural.

 The biotools enumerated by the authors are by no means exhaustive since environmental biotechnology is highly dynamic as well as futuristic. Those commonly practiced much elsewhere and very little if at all in Nigeria, which are being improved upon, are aquaculture treatment/management, biomonitoring, bioleaching, biocatalysis, biodetergent/biosolvent production, biofiltration, bioremediation, biomass fuel production, and so forth. Therefore, improvement in the quality of human, environment anywhere can be attained by applying any of the biotechnological tools suitable in such an environment. Nigeria has already adopted some such as bioremediation, biomonitoring, biofiltration, biofuel, and biocatalysis; however, their volume of their adoption is still very low. There is therefore need to adopt these biotools much and to look at ways of encouraging the local industries to adopt biotechnological tools in their production process so as to maintain the Nigerian environment.

 Developing Nations should instead of despairing on the damaged environment due to refuse heaps, mining activities, industrial production, and so forth embrace these practice (biotools) so as to improve their environment as well as to maintain and prevent continuous degradation of the environment. The Niger Delta regions of Nigeria had experienced continuous defacing of the environment due to crude oil pollution. Other nations have also had battered environments (water, soil, air) due to numerous industrial activities. Finding alternative ways of production, favourable to the environment as well as eco-friendly means of dealing with waste will go a long way in sustaining the environment.

## Figures and Tables

**Table 1:** Classification of biotechnologies. Modification from: Disilva [17].

Red	Medical
Yellow	Food biotechnology
Green	Agriculture
Blue	Aquatic
White	Gene-based industry
Grey	Fermentation
Brown	Arid
Gold	Nanotechnology/bioinformatics
Purple	Intellectual
Dark	Bioterrorism/warfare

**Table 2:** Environmental process and bioremediation procedures involved.

Environmental condition	Biosystem/microbes used	Bioremediation benefit
Waste water and industrial effluents	Sulphur-metabolising bacteria	(1) Microorganisms in sewage treatment plants remove common pollutants (heavy metals and sulphur compounds) from waste water before it is discharged into rivers or sea. (2) Production of animal feed from fungal biomass after penicillin production in penicillin industries. (3) Useful biogas (methane, etc.) production from anaerobic waste water treatment.

Drinking and process water	Organic degrading microbes (Bacteria, fungi, and algae)	(1) Reclamation and purification of waste waters for reuse and provision of portable recyclable drinking water for the public consumption and for livestock use. (2) Remove wastes for organic fertilizer agric use.

Air and waste gases	Bacteria, fungi	Biofilter application of pollutant purifying bacteria. Application of bioscrubbers, immobilized microorganism in inert matrix and nutrient film trickling devices for better air and gas purification. For example, bioscrubber-based system for removal of nitrogen and sulphur oxides from flue gas of blast furnaces in place of limestone gypsum process, and elimination of styrene from the waste gas of polystyrene processing industries by a fungi biofilter model.

Soil and land treatment	*Pseudomonas* spp., *Bacillus* spp., Fungi, *Rhodococcus*, *Acinetobacter*, *Mycobacterium *	Both in situ (in its original place) and ex situ (somewhere else) are commercially exploited for the cleanup of soil and groundwater. Use of microorganisms (bioaugmentation, ventilation, and/or adding nutrient solution (biostimulation) that is, petroleum decontamination, can involve use of plants (phytoremediation). Bacteria in association with roots of plants (Rhizobacterium), and so forth. Use of bioreactors for ex situ treatment with introduction of suitable microbes and environmental factors.

Solid waste	Bacteria, fungi, and so forth	Composting or anaerobic digestion of domestic and garden wastes helps in recovery of high-value biogas and useful organic compost without the toxic components. Free breakdown of solid waste by microbial biota for recyclable waste, an acceptable alternative to incineration.

**Table 3:** Biomarkers and their applications

Biomarker type	Uses	Reference
Chlorophyll content *Zea mays *L.	Detection of level of hydrocarbon contamination of agricultural soil	[11]

Sensitivity of *Nitrobacter *sp.	Based on the effect of crude oil on oxidation of nitrite to nitrate	[41]

*Azotobacter *sp.	Used in evaluating the effect of oil spill in aquatic environment	[42]

Algae/plant steranes and bacteria hopanes	Steranes formed as components of crude oil and hopanes used to determine the source rock that generated a crude oil	[43]

Ethoxyresorufin-O-deethylase (EROD) in fish in vivo	Indicates exposure of fish to planar-halogenated hydrocarbons (PAHs) by receptor-mediated induction of cytochrome P-450-dependent monooxygenase exposed to PAHs and similar contaminants	[44, 45]

**Table 4:** Bioethanol plants in Nigeria.

Name of Company	Plant location	Feed stock	Installed capacity (million litres/year)
Dura Clean	Bacita	Molasses/Cassava	4.4
AADL	Sango Ota	Cassava	10.9

**Table 5:** Proposed plants.

No.	Name of company	Project information	Budget
1	Jigawa, Benue, Anambra and Ondo States	Integrated bio-ethanol refineries and sugarcane farm	US$4 Billion
2	Nasarawa state	Integrated bioethanol refinary and cassava farm	US$27 Million
3	Casplex	Ethanol refinery and cassava farm	NA
4	Akoni	Ethanol plant	NA
5	Ekiti state	Integrated bioethanol refinary and cassava farm	US$100.7 Million

NA: not available.

Source: Agbola et al. [79].

**Table 6:** Environmental friendly application of enzymes.

Industrial sector	Description	Enzyme application	Reference
Fine chemical production	Biocatalysis using selectivity of enzymes for one of the enantiomers of a chiral molecule, that is, one enantiomer of a racemate is unaffected and the other enantiomer is converted into the desired, pure chemical	Hydrolases are most prominent enzyme used in production of fine chemicals by biocatalytic resolution	Schulze and Wubbolts [85]

Biopolymers/plastics	Enzymes or whole cell systems use sugars as feedstock for product manufacturing	Microbial/enzyme emulation of fossil fuel process	

Nutritional oil production	Genetically enhanced biomass (e.g., soybeans) to yield oil with improved properties, especially functional and nutritional quality	Increasing concentration of *β*-conglycinin, a seed storage protein	Harlander [86]

Ethanol production	Feed stock is cellulosic biomass (e.g., corn ears and stalks, wheat straw, or switchgrass)	Recent advances in cellulose enzymes have improved efficiencies	Knauf and Moniruzzaman [87]

Leather degreasing	Developing proteases for use in soaking, dehairing, and bating processes	Proteases from *Aspergillus tamarii* and *Alcaligenes faecalis* and loosen hair without chemical assistance. Alkaline protease produced from *Rhizopus oryzae* through solid-state fermentation dehairs the skins completely; use of enzymes for dehairing; baterial cultures have keratinolytic activity	Thanikaivelan et al. [88]

Biohydrogen production	H_2_ reactions catalyzed by either nitrogenase or hydrogenase enzymes	*E. coli, Enterobacter aerogenes*, and *Clostridium butyricum* use multienzyme systems. Can continuously produce H_2_ photochemically and nonphotochemically. Nitrogenase enzymes from *Rhodopseudomonas palustris* and *Rhodobacter sphaeroides* generate H_2_ under N-limited conditions	US Department of Energy, Office of Science [89]

Chemical/biological warfare agent decontamination	Enzymatic processes can speed the decomposition of organophosphate nerve agents and other warfare agents	Bacterial enzymes catalyze hydrolysis from bacteria genetically modified to express protein variants, for example phosphotriesterase and organophosphorus anhydrolase	Richardt and Blum [90]

Pulp and paper bleaching	Xylanase is applied before bleaching, replacing Cl-containing compounds in the first stage of the five-stage bleaching sequence. While rot fungus (*Phanerochaete chrysosporium*) degrades lignin in bioreactor wood chips injected with fungus and a growth medium, incubate for 2 weeks, followed by traditional chemical or mechanical processes	Enzyme replaces traditional Cl-addition. Biotechnology process reduces the amount of Cl-containing compounds by more than 10%. Bioreactor method reduces bleaching-related energy requirements by 40%, with concomitant pollution reduction	Roncero et al. [91]

Electroplating/metal cleaning	Enzymes make degreasing/metal cleaning. Fungi can be used to treat metal-laden waste	Proteases may be similar to those listed for leather degreasing *Aspergillus japonicus* used to sorp metal ions, for example, Fe (II), Ni (II), Cr (VI), and Hg (II)	Ahluwalia and Goyal [92]

Source: adapted from: [93].
